# Setting off on the right path: make your research regulatory relevant

**DOI:** 10.3389/frma.2025.1561964

**Published:** 2025-06-16

**Authors:** Anna Pohl, Elise Morel, Eric A. J. Bleeker, Sean Kelly, Rachel Smith, Claus Svendsen, Thomas A. J. Kuhlbusch, Elisabeth Heunisch

**Affiliations:** ^1^Hazardous Substances and Biological Agents, Federal Institute for Occupational Safety and Health (BAuA), Berlin/Dortmund, Germany; ^2^UK Centre for Ecology and Hydrology (UKCEH), Wallingford, United Kingdom; ^3^TEMAS Solutions GmbH, Hausen, Switzerland; ^4^Centre for Safety of Substances and Products, National Institute for Public Health and the Environment (RIVM), Bilthoven, Netherlands; ^5^Nanotechnology Industries Association Aisbl (NIA), Brussels, Belgium; ^6^UK Health Security Agency (UKHSA), Didcot, United Kingdom; ^7^Nano Particle Process Technology (NPPT), University Duisburg-Essen, Essen, Germany; ^8^Center for Nanointegration Duisburg-Essen (CENIDE), University Duisburg-Essen, Essen, Germany

**Keywords:** regulatory safety testing, OECD test guidelines, method development, method validation, standardization

## Abstract

Scientifically well-established methods do not automatically get used in regulations. Even when there is an urgent need for regulatory relevant test methods, methods need to pass through a so-called standardization process. This involves following specific agreed processes, which define the timeline and requirements (e.g., validation, documentation, approval) before the method can be integrated in regulatory oriented standards or Test Guidelines from the Organization of Economic Cooperation and Development (OECD). The process is often seen as too complex or too resource (and time) consuming by the scientific community, which inhibits method developers from translating their scientific methods and protocols into standards or OECD Test Guidelines. Numerous incentives exist for scientists to be (more) active in the standardization process and allow regulation to keep up with new scientific developments. These include an increase in research impacts, an expansion and diversification of the international expert network, and an access to more fundings. This paper shows scientists how to reach such outcomes, by providing guidance on how to navigate successfully through the standards and OECD Test Guidelines development processes. Especially the requirements for method validation, which is a prerequisite in this process and common across the different standardization bodies. For further details and insights, readers are invited to consult the various freely available resources generated by the NanoHarmony EU project. These are compiled in the OECD Test Guideline Process Mentor (https://testguideline-development.org/). The active participation of scientists along the entire process toward standards and OECD Test Guidelines is key. Only then can their methods be expanded into a wider, regulatory application toward a safer world.

## 1 Scientific knowledge is key for much needed up-to-date OECD Test Guidelines and standards

Good science does not automatically get used in regulatory work. A significant portion of scientific publications are not very useful for the purpose of the development of standards and Test Guidelines (TGs) from the Organization of Economic Cooperation and Development (OECD).^1^ One main reason is that scientists who develop test methods are usually not familiar with the requirements to design, validate and standardize cost effective and broadly applicable methods that provide sufficient information to allow regulatory decisions.

In this publication, the term standard is generally used to refer to standards issued by standardization bodies [e.g., from the International Organization for Standardization (ISO)^2^]. A subset of these are technical standards, covering chemical and material characterization and safety testing that are required for regulatory compliance of substances and mixtures, and these standards are the main focus here. We refer to these as “harmonized standards” when it is necessary to differentiate them from the wider range of available standards. The differences between standards and OECD TGs are highlighted in Table 1.

**Table 1:** The key aspects of standards and OECD Test Guidelines, including the main drivers for their development, are listed and the differences between standards and OECD Test Guidelines are highlighted.

Standards^*^	**OECD Test Guidelines**
Standards are mainly technical specifications, adopted by a recognized standardization body, for repeated or continuous application, with which compliance is not compulsory (EC, 2012). A subset of standards are harmonized standards that aim to provide a technical foundation to meet the essential requirements set out in regulations.	OECD Test Guidelines are recognized internationally as standard methods for safety testing. They are an integral part of the Mutual Acceptance of Data agreement. OECD Test Guidelines are used to support chemical safety regulations in many countries.^3^ Each Test Guideline provides sufficient detail for chemicals to be tested in the same manner in laboratories around the world.
Mainly market need _ More industry driven _ Communication among different stakeholders _ Participation via national standardization bodies	Regulatory need _ More government driven _ Data gathering and enforcement of legislation _ Participation via the national delegation or official institutional partner
Often specific for an industry, a technology, a product, a process, or a material. _ Applicable for performance and quality control, for environmental and health safety, for sustainability and ethical best practices.	Structured along endpoints within the sections physical-chemical properties, effects on biotic systems, environmental fate and behavior, health effects, and others.
The development process usually takes about 3 years	The development process can take 2–5 years on average
Purchase for (small) fee	Public access

^*^The information relates primarily to ISO standards and their development, however, other standardization bodies work in a similar manner, so the points are widely applicable.

Standardization is important for a global exchange of knowledge and accelerating science. It forms part of the common language needed for such global exchange, not only in science, but also in markets. Global organizations like the ISO and the OECD are key players in providing harmonized and standardized methods to establish common language and methods and ensure globally accepted reliable and good quality data on physicochemical characterization and safety of chemicals.

The European Union views standardization not only as a means to facilitate the industrial transition and acceptance of innovative methods and products (e.g., Strategy for Industrial leadership on Advanced Materials; EC, 2024) but also as a way to foster safety and environmental protection by providing tools to demonstrate compliance with EU laws. With the Green Deal and other underlying strategies as for example the Chemical Strategy for Sustainability (CSS; EC, 2020), or the Safe-and-Sustainable-by-Design (SSbD) recommendation (Caldeira et al., 2022), Europe aims for a transition toward a sustainable future, using harmonized standards and OECD TGs as important tools for risk assessment and regulatory decisions (e.g., market entry). Such tools are already incorporated in European legislation, most notably in the European Test Method Regulation (EC, 2008) that provides harmonized methods for regulatory (safety) testing in Europe. This regulation has been regularly updated to ensure methods are updated with the latest scientific insights (e.g., on how to test new materials), or incorporate new regulatory requirements (e.g., on new toxicological endpoints, or minimizing test animal use). This illustrates that science and regulation co-evolve and influence each other. Scientists may identify (a need for) new regulatory endpoints (e.g., endocrine disruption, developmental neurotoxicity) or more effective test methodologies, which may partly be triggered by innovations in science/industry toward more complex substances/materials.

As developments in innovations tend to speed up, industry, risk assessors and regulators need more and faster development of harmonized standards and OECD TGs to keep pace with innovation and to cover regulatory risk assessments. Testing with OECD TGs under good laboratory practices (GLP) leads to comparable results and international acceptance of data avoiding double testing as stated in the OECD Mutual Acceptance of Data (MAD) agreement. This can reduce the number of animal tests, reduce costs, minimize trade barriers and enable international comparison. As a result, this saves governments and industry currently ~€ 309 million a year (OECD, 2019), a figure that is expected to rise as the results of more environmental health and safety projects become available in the coming years. In addition, harmonized standards and OECD TGs not only enable regulation but also offer benefits to academic research (e.g., in harmonized test methods facilitate comparisons of scientific results).

Participating in the field of standardization and harmonization and hence advancing scientifically developed methods toward harmonized standards and OECD TGs can lead to a better visibility of scientists in the field of safety research, a broader application and social impact of their methods and the satisfaction of contributing by their work to a safer and more sustainable world. Standardization and harmonization of new methods, however, is often hindered by various hurdles that were identified by the project NanoHarmony, a European coordination and support action project. With this paper we aim to encourage scientists not already active in the regulatory and standardization area, to advance their methodology to a standard or an OECD TG. The paper gives guidance on how to achieve this, while showing benefits for the scientific community.

## 2 NanoHarmony analysis of the OECD TG development process

Actors in the development process of harmonized and standardized methods, or those that may want to become active in the process, may perceive difficulties that they did not expect. In the NanoHarmony project such hurdles in the development of TGs were identified together with recommended solutions. To identify the hurdles encountered by different stakeholders when contributing to OECD TG developments, NanoHarmony consulted developers of OECD TGs, regulators and users of OECD documents [TGs or Guidance Documents (GDs)] via a survey, interviews, workshops, and meetings (NanoHarmony Deliverable 3.2^4^). The workshops and webinars organized by NanoHarmony on a regular basis formed further opportunities to identify and discuss hurdles and challenges in the process and collect input on what the different stakeholders may need to facilitate the process. These exchanges included stakeholders from academia, industry and service providers, standardization bodies, governmental agencies, the general public and non-governmental organizations. Although having reached out to a broad range of different stakeholders, the participation of experts associated to small and medium size enterprises (SMEs), contract research organizations (CROs), non-governmental organizations (NGOs) and policy makers was underrepresented in the NanoHarmony survey and webinars. Furthermore, it is often difficult to obtain the opinion of the entire stakeholder group or even what could be regarded as a statistically relevant sample, and the information gained is more of an individual opinion by the experts. However, these opinions are still useful in helping identifying challenges to be addressed to encourage greater participation in the OECD process.

On top of this, NanoHarmony built on the gathered experiences of project partners during the development of OECD documents. Within NanoHarmony, eight OECD projects were further developed in different sections within the OECD Test Guideline Program (TGP).^5^ Toward the end of NanoHarmony, the challenges identified and the lessons learned were shared in in-person meetings with the OECD and policy makers to further discuss the outcomes and potential ways forward. Recommendations for the scientific community, further elaborated upon in the paragraphs below, are the results of all these exchanges with a huge number of international experts and stakeholders on their experiences with the OECD TG development process. Getting an opinion from the OECD itself, however, is impossible since the decisions by the OECD are result of negotiated agreement between Member Countries. By reaching out to international experts outside the EU, by including associated partners within the EU-funded project and by interacting with the OECD Secretariat, NanoHarmony widened its EU project perspective to an international view.

**Figure 1:**
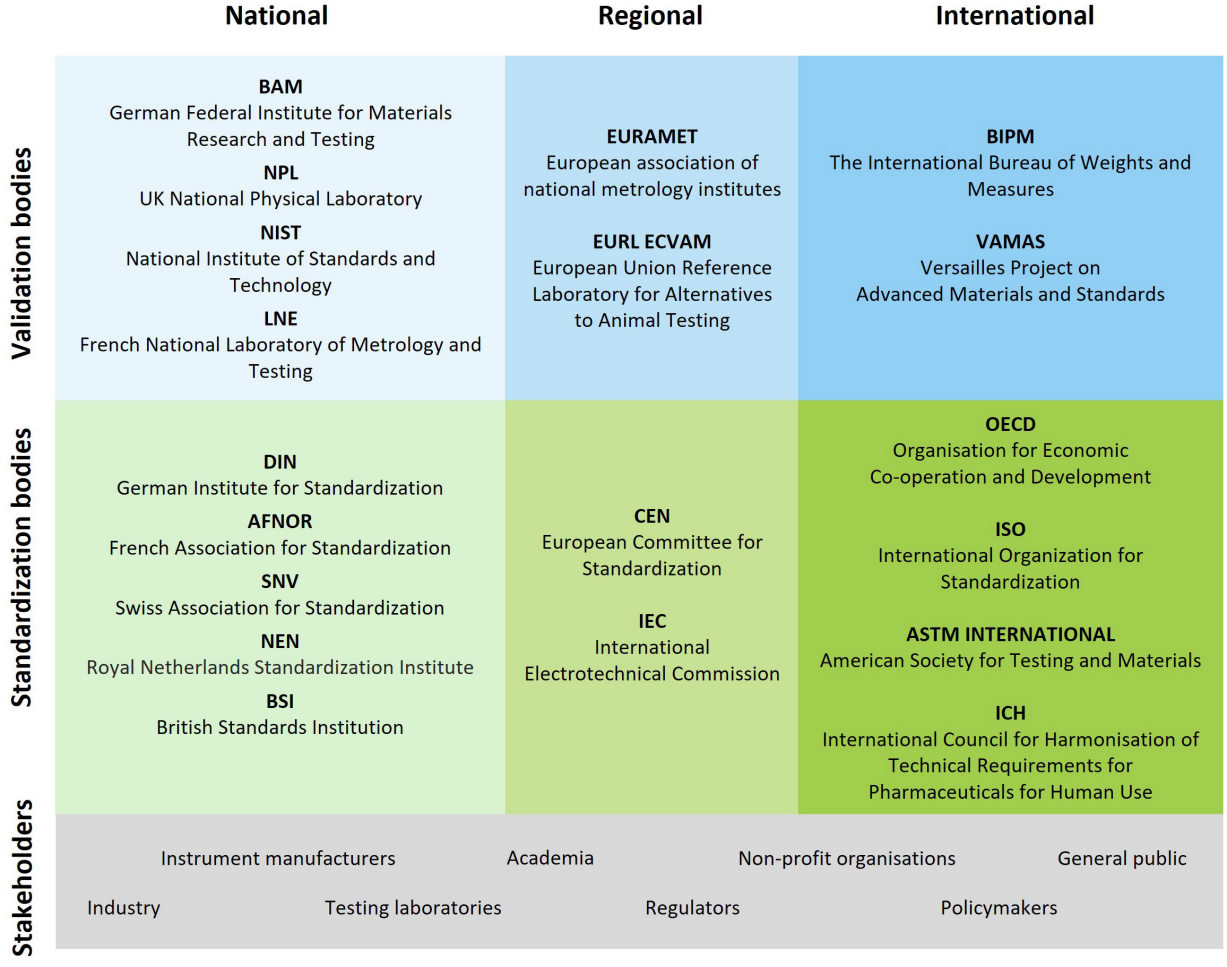
Schematic overview showing the landscape of validation and standardization bodies at a national, regional, and international level. The figure lists examples of key players within the field of standardization and harmonization. Furthermore, the different stakeholder groups involved in standardization and harmonization efforts are listed at the bottom of the figure since they build the fundament for the development of standards and OECD Test Guidelines. The list is not meant to be comprehensive.

While NanoHarmony focused on the OECD TG development process, most of the issues identified are also valid for processes in other standardization organizations. There is significant cooperation across the standardization bodies to avoid work duplication and ensure national, regional and international standards are consistent (see Figure 1 for examples of validation and standardization bodies). Besides these close collaborations there are also sometimes difficulties observed in consensus finding. Since the processes in the different standardization bodies are quite similar, NanoHarmony did not go further into detail in the differences and did not try to extract those parts of the process that lack similarity. The material developed by NanoHarmony informing about the OECD TG development process could be expanded to fully cover the processes for the other standardization bodies. In addition, monitoring of the overall impacts of the NanoHarmony legacy materials on the contribution of the scientific community in the standardization field could be explored to identify how to further expand the content and capture the full range of standardization activities.

## 3 How to be successful in the development of standards and OECD TGs

As a result of the process analysis performed by NanoHarmony it was evident that scientists play a key role within the development of test methods and that there are specific aspects scientists should consider in order to be successful in the development of standards and OECD TGs.

### 3.1 Benefits for scientists to contribute to standardization and TG development

As a first step, scientists need to know that they can provide valuable input into the development of standards. Even where awareness exists, there is often a challenge for scientists to see the benefits they may have in working on a TG or standard. The very nature of academic research can make it challenging for academic scientists to engage in the TGs or standards development process. Career progression in universities is driven through publications and funding, which encourages the development of new cutting-edge projects rather than continuing to push a developed method through the standards or TG development pipeline. The formal processes by which OECD TGs or standards are developed and approved are generally very time and labor consuming (see Section 3.6). This is not compatible with short-term contracts experienced in academic research.

However, there are already some incentives for scientists to consider taking this step. Firstly, it is a good opportunity for scientists to develop societal impact through their work being used in regulatory and industrial testing. Apart from showing the practical application and usefulness of their work, the increased use of the method will lead to higher citations of publications. Secondly, the development process allows the scientists to expand their network and work with regulators, industry and policymakers. This can help scientists in identifying new areas for their research focus and potential future collaborators. Thirdly, for junior scientists working on developing TGs or standards gives them an experience of working outside of academia, helping prepare them for following other pathways into a scientific career. Finally, there has been an increasing number of research project calls asking for the implementation of research results in standardization and linking with policy and industry.

The update of TGs and harmonized standards is normally triggered by new method and material developments or new (toxicological) insights. Further incentives for new developments are policy goals, as for example moving away from animal testing and using alternative testing methods. Therefore, scientists need to get an overview of possible standardization bodies that could be approached and of the ongoing projects and standardization needs. Information on ongoing standardization projects is published on the websites of the standardization working groups and committees. An overview on the OECD TGP can for example be found on its webpage, including the current work plan.^6^ Activities at ISO are published on the Online Browsing Platform^7^ and ASTM activities can be screened using their repository.^8^ The Versailles Project on Advanced Materials and Standards (VAMAS) provides information on ongoing interlaboratory comparison on its webpage.^9^

### 3.2 Scientific projects are essential to support TG and standard developments

Scientific research projects are important starting points for method development. Projects can also be (partly) dedicated to specific method development to support innovations and answer to regulatory questions or needs. Some of the needs to address may already be clearly indicated in calls for projects (e.g., EU funded projects), but projects may also identify needs themselves or respond to needs put forward by others.

As an example, the EU-project REFINE put out a white paper that identified regulatory information needs for nanotechnology-enabled health products, i.e., focusing on needs in the medical field (Halamoda Kenzaoui et al., 2019). For needs on nanomaterials and other advanced materials in the area of industrial chemicals the Malta Initiative identified needs for updating or developing test methods (Malta Initiative, 2024).

EU projects like Gov4Nano^10^ and NanoHarmony^11^ have shown that projects can also closely support specific projects that are ongoing in OECD. While the actual writing of the OECD documents falls under the responsibility of a specific Member Country in OECD's Working Group of National Coordinators of the Test Guidelines Programme (WNT), the scientific basis was developed by these scientific projects (Heunisch et al., 2022).

These examples illustrate that scientific research is essential in addressing and identifying needs for regulatory test method development. Furthermore, it should be emphasized that innovation in test methods is a continuous process (e.g., as highlighted in the Malta Initiative Priority List; Malta Initiative, 2024) and newer projects are picking up this challenge (e.g., MACRAMÉ, nanoPASS, iCARE, CHIASMA).

To enable an exchange on TG development and the needs for nanomaterials and other advanced materials, NanoHarmony started a yearly online workshop on standardization and harmonization that is currently continued by the EU projects MACRAMÉ, iCare, and nanoPASS.

### 3.3 Funding is required from the beginning to the end

There can be challenges in identifying funding accessible to aid OECD TGs and standards development. Accessibility of funding also depends on several factors including, the phase of the development process in which funding is required, the country the developer is based in, and the reasons why the funding is needed. It is an unfortunate fact that development of both TGs and standards can often be impacted by the unavailability of funding, either because there is no funding available, or the funding available is not sufficient to cover the required costs. The pre-OECD stages of TG development, when new guidelines are conceived and scientific advances are still being made, are often financed through research funding. For example, the European Union in its Horizon Europe program encourages projects to exploit relevant results into TGs and standards. However, the development processes for these are often longer than the project funding available and it is unusual for research projects to be able to cover the cost of the later commenting phases in TG or standard development.

Some national authorities do make funding available to allow researchers to participate in the technical developments of TGs and standards, but this is very dependent on the country the developer is based in. The main advice that can be offered to TG and standards developers is to discuss this situation with their relevant national contact. In the case of the OECD this would be their National Coordinator.^12^ For standards, it would be their national standardization body.

### 3.4 Building contacts with the OECD working groups and standardization bodies is important

**Figure 2:**
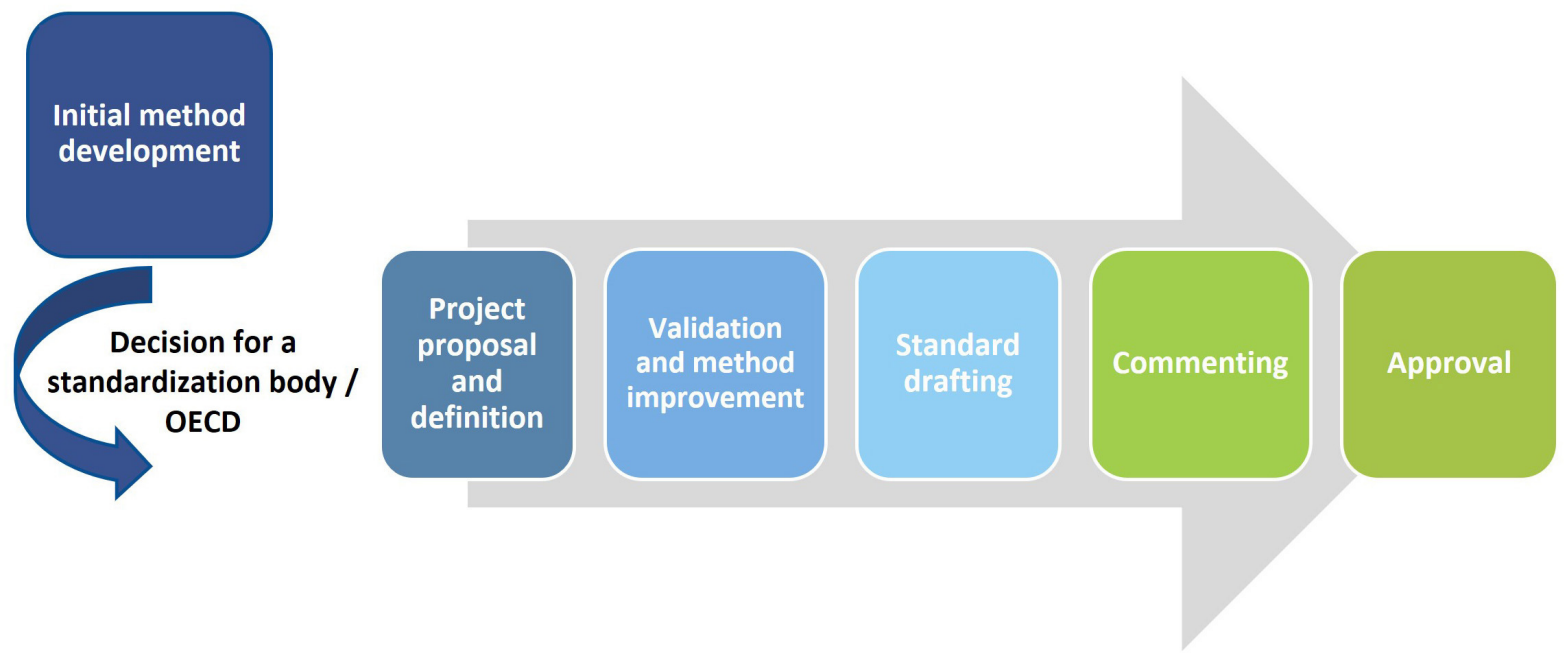
Schematic overview of the development process toward standards and OECD Test Guidelines. This formalized process includes the initial method development followed by a decision for the respective standardization or harmonization body. Once this is accomplished, the process of the standardization or harmonization body needs to be followed. Key steps within this process are the proposal of the project, the validation of the method, the drafting of the standard and the commenting rounds followed by the successful approval of the new or updated Standard or OECD Test Guideline.

To get insights into regulatory needs, scientists need to get into contact with regulators and the national representatives within the standardization bodies and OECD. This can be established using scientific projects, through participation in stakeholder exchange events or through direct contact. The National Coordinators are pivotal in the development of TGs. At OECD, they oversee the TGP and discuss progress on TG developments in their annual meetings in Paris. This also includes identifying needs for new developments and agreeing on the approval of new or updated TGs. The National Coordinators represent policy and regulatory authorities in Member Countries at the OECD TGP and coordinate activities on this in their own country. They nominate experts and scientists from research and regulatory areas to work together on developing tools and guidance. In this role, they generally form a first entrance for scientists into the OECD processes, not least because they are the only ones that can propose a new project for inclusion in the OECD TGP. This shows that it is beneficial for scientists to build contact with their own National Coordinators (and other representatives in OECD working groups). Furthermore, in overseeing the TGP, National Coordinators see many different approaches and participate in relevant discussions. This allows them to guide TG developers through the process. They can support the identification of relevant stakeholders to be involved in each part of the standardization process (Figure 2), and more generally point the TG developers toward the relevant information. Based on the personal network that National Coordinators build as part of the TGP community, they are well equipped to coordinate the process when they represent a lead country for a TG in development. In coordinating, they can also help ensure effective and efficient communication with all relevant stakeholders during the process and ensure that discussions and decisions are captured and shared.

### 3.5 International exchange and collaboration is key

Close collaborations and exchanges with stakeholders from different backgrounds, including academia, industry, non-governmental organizations and policymakers are important in all steps of the standardization or harmonization processes. Standards and OECD TGs are made by consensus of all stakeholders involved in their development. It ensures that standards and TGs represent the best available practices in different sectors. At the level of international standardization and harmonization institutions such as ISO and OECD (Figure 1), stakeholders involved in these processes are designated from member countries, associated countries and partner institutions and thus span the world. This creates a challenge for scientists to ensure that they can be effective in communicating their work with experts with different backgrounds and from different countries. Although the needs of reaching out to different stakeholders will be different in different steps of the process, a good national and international network is beneficial in all steps of the process to address this challenge.

Early on in the development process, it is helpful to identify experts from different backgrounds and countries that can form an expert group that is consistent with the breadth of the standardization or harmonization project. The expert group should provide an appreciation of the contextual circumstances for all the study aspects, as well as an understanding of the regulatory applicability of the project in different sectors and regions. For example, in some countries, it may be difficult for users to have access to certain equipment or consumables and experts from these countries should highlight such limitation to ensure a broad applicability of the harmonized standard or TG under development. At the later stages of a harmonized standard or TG development, close collaboration between regulators and academic scientists is needed so the commenting rounds can be managed effectively, and developers can address both scientific and regulatory comments.

Establishing a network and ensuring exchange and collaboration can be supported by National Coordinators at OECD, and other relevant projects or initiatives (e.g., NanoHarmony, Malta Initiative^13^). If needed, the National Coordinators can also facilitate clear communication and enhancing understanding of the different stakeholders.

### 3.6 Knowing the process to establish a standard or an OECD TG is a prerequisite

In order to be successful with the transformation of scientific developments into standards and TGs it is pivotal to know the process of standardization and harmonization. Each standardization body has its own process to establish a standard. Getting familiar with the essential steps within the process, the strict deadlines and the information requirements is key. For in-depth details on the OECD TG development process, it is recommended that the NanoHarmony Process Mentor^14^ is consulted.

Prior to the first step in the official standard and TG development process, the method needs to have reached a certain level of maturity. To be picked-up for standardization, a test method must be relevant and reliable, commercially available and have demonstrated a strong potential for application in several case studies (i.e., exploited by as many end-users as possible). As a general guideline, for a method to be considered for standardization, it should be substantiated by a significant body of work, typically reflected in peer-reviewed publications.

One should note that standardization bodies will favor competitiveness between technology and material suppliers in order to avoid commercial monopolies. It is thus required to evaluate the equipment and consumables associated to a test method and ensure their broad availability across the world when starting to think about its standardization. Central steps of the process toward standards and TGs are initial method development, the selection of the standardization body to approach, project definition and project proposal, validation and method improvement, standard or TG drafting, commenting and approval (Figure 2).

The role of scientists within this process is greater in the following steps: method development (e.g., by providing SOPs), validation (e.g., by participating in validation studies), method improvement (e.g., by providing scientific knowledge) and standard or TG drafting. Within all the different steps scientists can and should get involved to bring their scientific developments and results to the regulatory arena. However, in the context of ever-evolving science, scientists should abandon the idea of producing the best possible method that covers everything and aim to limit their protocol development to the essential minimum that provides only the necessary information to allow regulatory decisions. Standards development must focus on designing cost-effective protocols that are easy to follow and easily transferable to different laboratories in different geographical regions.

### 3.7 Validation of test methods is essential

Validation is a central element of the development process of standards and TGs since it is key for enabling regulatory use of test methods. Validation establishes confidence and trust in the methods as well as the data generated by using these methods. OECD defines validation as “The process by which the reliability and relevance of a particular approach, method, process or assessment is established for a defined purpose” (OECD, 2005). This definition is essential in the OECD TGP that ensures international acceptance of test methods under the OECD's MAD framework. Validation is a process that starts after many scientists will consider that their method development is finished. It, however, often leads to new insights and fine-tuning. To start the process of validation, the method should already have a certain maturity in terms of having limited proof of its reliability and relevance based on some pre-validation experiments. For proving the relevance of a test method for regulatory purposes, validation studies should provide insights into the applicability domain, usefulness, and limitations of the test method.

For determining the applicability domain, the use of standard materials or benchmark chemicals are needed. Such materials can be identified in reference material databases (e.g., NIST,^15^ COMAR,^16^ LGC Standards,^17^ Certified reference materials catalog of the JRC^18^ and JRC nanomaterials repository^19^). To be validated in the standardization context, a method must generate repeatable results with an acceptable precision and uncertainty when independent samples are analyzed by the same laboratory (i.e., intra-laboratory comparison), and generate reproducible results when samples are analyzed by several laboratories (i.e., inter-laboratory comparisons). When performing intra- and inter-laboratory comparisons several aspects should be considered:

- Use of different materials (e.g., chemical composition, morphology, physical-chemical properties, etc.) enables a broad validation of the method.
- The international participating laboratories should be independent from each other.
- Intra- and inter-laboratory comparison studies can take a long time and require effort in terms of resources, laboratory work and organization. This should be reflected in the project schedule.
- Documentation of the decisions and discussions in the project helps to find consensus and to inform all potentially new contributors. Additionally, it helps to streamline the commenting process.
- Validation studies should be tailored to the specific requirements of the scientific field, the context and the purpose.
- Results from validation are compiled in validation reports and generally supported by peer-reviewed publications and technical documents.

Since validation studies can be different depending on the scientific field, specific guidance for validation is available for some topics at OECD level:

- OECD GD No. 34 on validation and international acceptance of new or updated test methods for hazard assessment is currently under revision. It provides information on various aspects of validation (OECD, 2005).
- For the reliability of *in vitro* methods, OECD GD 211 was applicable to the different types of *in vitro* test methods (OECD, 2017). A more conceptual, overarching guidance, analogous to GLP, has been published with the OECD Guidance Document on Good *In Vitro* Method Practices (GIVIMP; OECD, 2018).
- The OECD Series on testing and Assessment document No. 329 provides information on available concepts and guidance related to Integrated Approaches to Testing and Assessment (IATA; OECD, 2020).
- OECD Series on testing and Assessment document No. 331 informs on the characterization, validation and reporting of Physiologically Based Kinetic (PBK) models for regulatory purposes (OECD, 2021).

Various organizations support validation, e.g., French National Metrology Network, European Metrology Networks (EURAMET), EU Reference Laboratory for alternatives to animal testing (EURL ECVAM), VAMAS at national, regional or international level (see Figure 1) for the landscape of validation and standardization bodies). An example to further the validation of test methods is PEPPER.^20^ This public-private platform aims to organize and fund scientific research and testing to pre-validate methods for endocrine disruptors characterization.

## 4 Practical support for scientists developed by NanoHarmony

**Figure 3:**
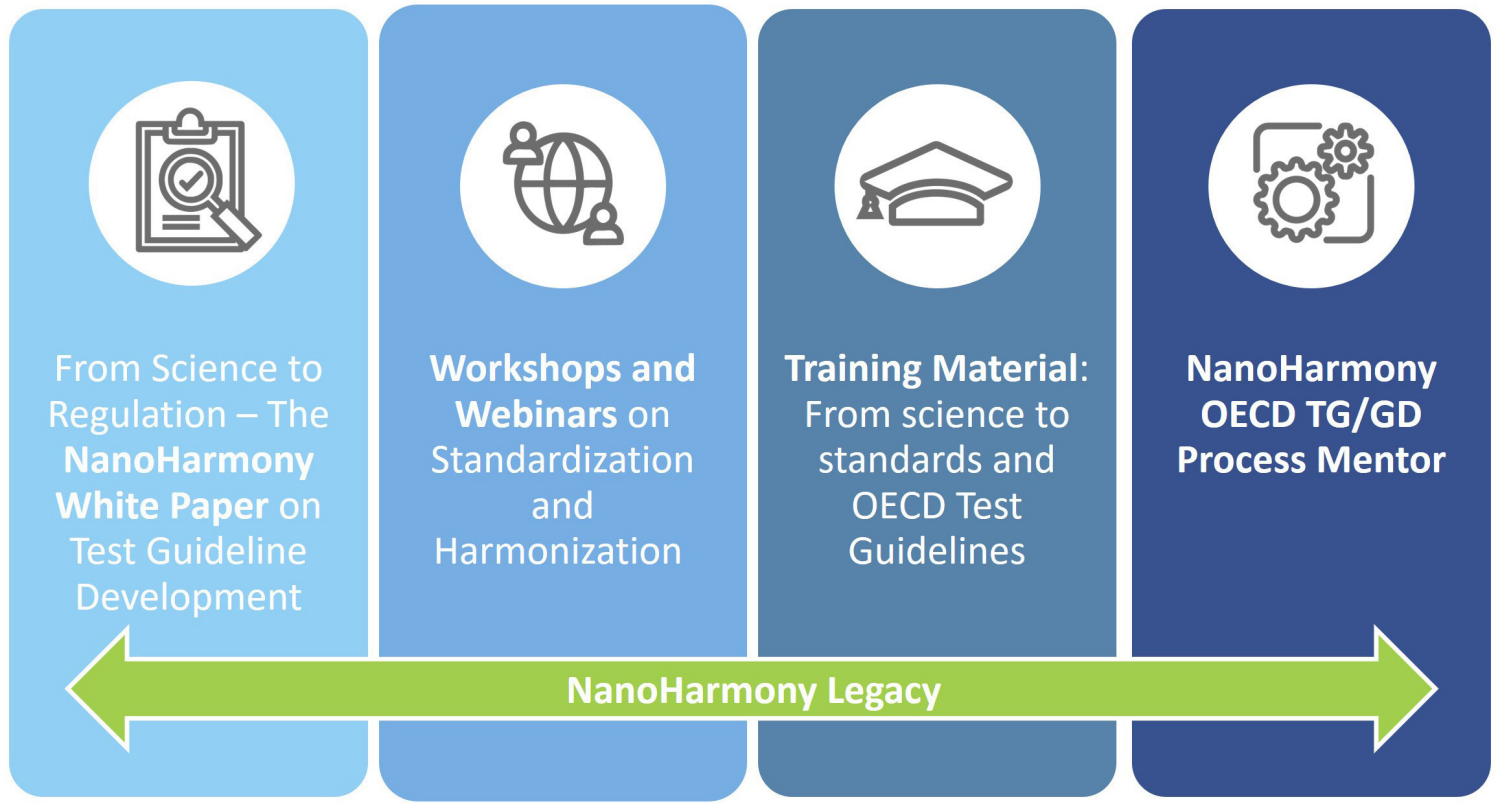
The EU project NanoHarmony generated several legacy items that are available beyond the project life time: The White Paper highlights the need for internationally agreed methodologies for testing and characterizing chemicals and advanced nanomaterials to protect human health and the environment, the recordings of Workshops and Webinars provide various insights into the pathway toward OECD Test Guidelines, the Training Material can be downloaded and used for further training and education on the OECD Test Guideline development process, and the online tool NanoHarmony OECD TG/GD Process Mentor guides interested people interactively through the development process.

Having identified and worked to resolve some of the barriers that prevent or slow scientific developments from being used for regulatory purposes, the NanoHarmony project has put in place some practical support mechanisms to aid scientists (Figure 3). The support offered by NanoHarmony will by means of its legacy items continue beyond the lifetime of the project. These legacy materials, while developed in context of nanomaterials, will be useful for anyone interested in OECD TGs and their development process, regardless of the test substances of concern, as the OECD process is the same for chemicals and advanced materials.

### 4.1 White paper

A cornerstone of NanoHarmony's legacy is the White Paper entitled “From Science to Regulation”.^21^ This central document outlines the necessity of international agreement on methodologies for testing and characterizing chemicals and advanced nanomaterials to protect human health and the environment. The OECD TGP, as emphasized in the White Paper, plays a vital role in ensuring that these materials are safe and sustainable. The White Paper makes eight recommendations to enhance the effectiveness of the TG development process, moving new methods from science to regulation more effectively. It is notably recommending to OECD Member Countries to encourage the essential scientific input into the process and to ensure the necessary funding.

### 4.2 Workshops and webinars to inform on standardization and harmonization

NanoHarmony ran a range of webinars and workshops during the project, which have been collated into an easily accessible and open library with recordings of 13 events held during the project lifetime being made available.^22^ The recordings provide various insights into the pathway toward OECD TGs, solving barriers in the process, and specific data requirements at all steps of the OECD TG development. Some of them were realized together with the EU-funded project NANOMET. They also provide an opportunity for people to hear from experts about their journeys through the OECD TG development process. The EU-funded project MACRAMÉ together with the projects nanoPASS and iCARE continue the journey and organize annual Harmonization and Standardization Workshops.^23^

### 4.3 NanoHarmony training material: from science to harmonized standards and OECD TGs

Offering a low-level introduction to the topic of standards and OECD TGs, the NanoHarmony Training Material^24^ is aimed at people with little or no knowledge of the OECD and its processes. The NanoHarmony Training Material can be used as a self-guided introduction to the OECD development process, as well as by educators looking to incorporate this topic into their teaching. For scientists, from postdocs working on a project, to scientists working in or coordinating research projects, the Training Material can provide a way to help support the transfer of knowledge from science to regulation and can help people interested in understanding how and why to transfer their research outcomes into TGs.

Four independent modules have been developed and build in complexity from introducing the importance of standards and harmonized methods, to the OECD and its structures and the whole process of developing OECD documents. They can be combined as required and allow the creation of presentations of different lengths and detail, depending on the requirements of the presenter or knowledge of the audience.

### 4.4 NanoHarmony OECD TG/GD Process Mentor

**Figure 4:**
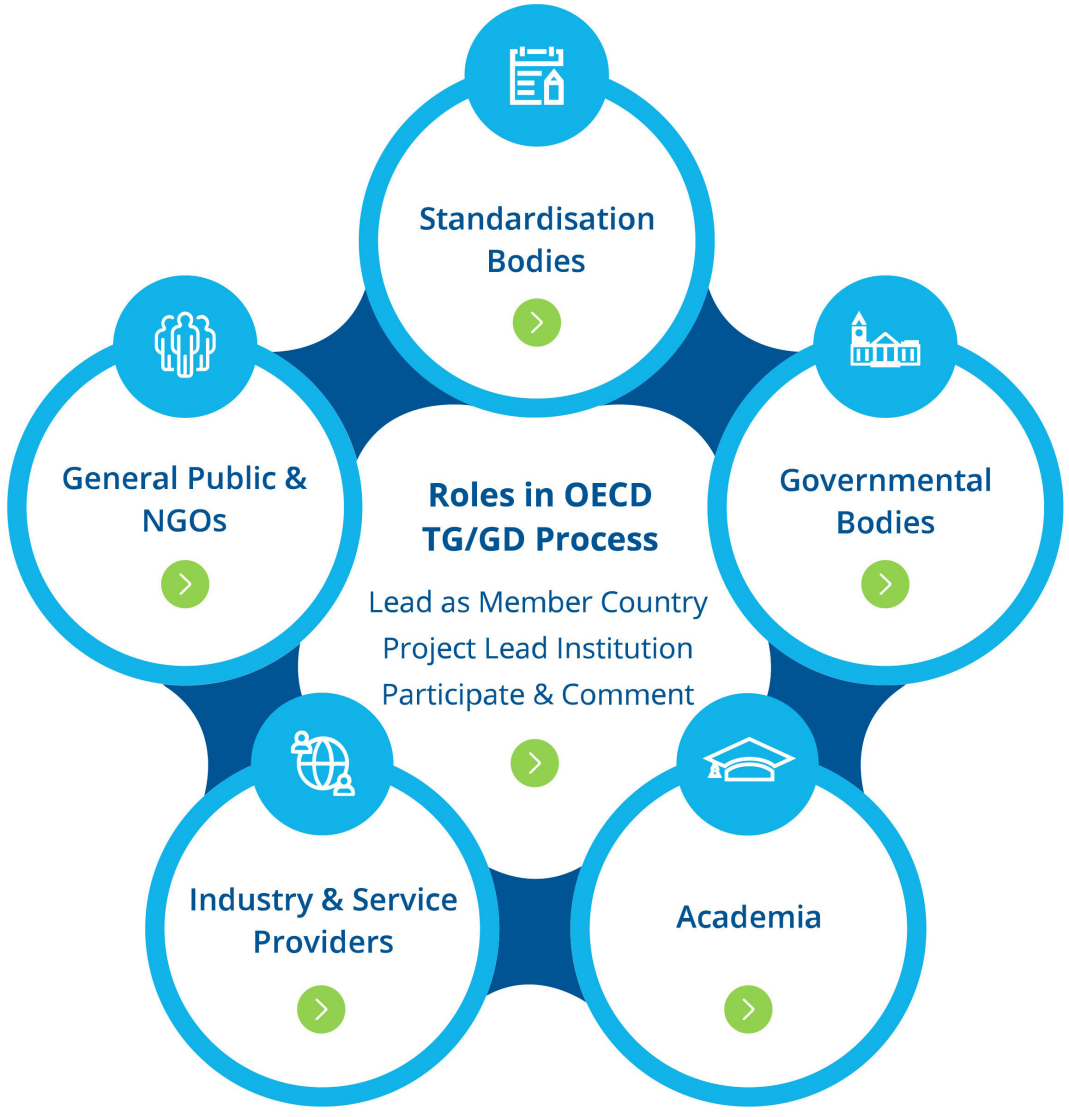
Schematic overview of the key stakeholder groups involved in the development process of OECD Test Guidelines. Further details on the roles of these groups are provided in the NanoHarmony OECD TG/GD Process Mentor.

The NanoHarmony Process Mentor is an interactive online tool that guides people through the development process for OECD TGs and GDs (Figure 4). It highlights the role played by key institutions, such as industry or governmental bodies and their incentives to participate in the OECD process. It provides guidance and key tips on developing or updating TGs, such as when to start certain processes, who needs to be involved and when their involvement is needed. It can be used by anybody but is especially aimed at scientists who want to or, due to funding body requirements, need to develop TGs. It complements the existing OECD guidance on the development of TGs (OECD, 2002) by providing both an intuitive visual guide through the OECD process, as well as bringing together practical advice about what needs to be achieved at each stage of the development process and the required next steps. Although developed as part of a nanomaterial dedicated project, the majority of the NanoHarmony Process Mentor is relevant to anyone interested in the OECD TG process from a chemicals or advanced materials perspective.

The NanoHarmony Process Mentor can be navigated in a number of ways:

- **By role** in the OECD process (lead as Member Country, project lead institution, participate and comment).
- **By stakeholder type** (including industry and service provider, academia or governmental body).
- **By phase** in the OECD process (such as pre-OECD phase or project definition at OECD).
- **By process** (including financial support, OECD Expert Groups or commenting aspects).

It also contains a number of useful resources, such as a FAQ, a glossary and a link to the different NanoHarmony legacy items. For scientists interested in learning more about how to develop their research into a TG or GD, moving through the phases in the OECD process is the most intuitive way to understand the steps one needs to go through and to get advice from TG developers and standardization experts.

## 5 Discussion and conclusion

New scientific developments of methods and materials are essential to approach major global challenges such as the safety of products and materials, the protection of humans and the environment and the reduction of resource consumption. The development of new materials (e.g., advanced nanomaterials) with outstanding functionalities and safe properties as well as the development of new test methods triggers the development of harmonized standards and TGs for safety testing. These new methods often are comprised of *in silico, in chemico* and *in vitro* approaches that facilitate high throughput screening for safety testing and that reduce the need for animal testing. Adverse Outcome Pathways (AOPs) and IATAs are seen by the OECD as tools to standardize the data collection and reporting when data are produced by New Approach Methodologies (NAMs). Thus, scientific inputs in the field of AOPs and NAMs are contributing to a more effective risk assessment and further the global acceptance of these methods. These developments should prioritize regulatory needs and include adequate demonstration of the reliability and relevance of their outcomes for the endpoint of concern. A list of priorities for the adaption and development of OECD TGs and GDs for nanomaterials and other advanced materials is published in the Malta Initiative Priority List (Malta Initiative, 2024). Other research areas where scientific contributions are needed to support harmonized standards and TGs developments can be identified following working plans and activities of key standardization bodies (e.g., OECD annual workplan^25^). Currently promising areas appeared to be NAMs, omics,^26^ characterization of complex materials and matrices (e.g., Xie et al., 2022; Friedrichs et al., 2025), especially along the life cycle of (products containing) advanced materials (e.g., Subramanian et al., 2023).

Active participation of scientists within standard or TG development ensures that scientific advancements are picked up by regulators and industry. On a more personal level, it allows individual scientists to increase the visibility of their scientific expertise, the wider societal impact of their work and can boost citations of papers when these are used for TG and standard development. The possibilities for expanding the scientific network to a community beyond the pure scientific field can also be seen as an advantage. Such communities could include industry, testing laboratories, NGOs, or regulatory bodies.

Key aspects to consider in order to be successful with the development of standards and TGs are to know the differences between standards and OECD TGs, to understand the main aspects of validation studies, to be in contact with the diversity of stakeholders acting in standardization or harmonization and to follow the defined steps and deadlines of the development process. The development process is not only scientific but also about finding consensus between various stakeholders and especially all member countries. A harmonized standard or OECD TG is not only about delivering the best scientific data, but needs to be simple enough to be applicable world-wide and deliver sound data for regulatory purposes. As the scientific sound test method and data is the core of the TG and standard development, a scientist can contribute in various different ways to the development of new harmonized standards and OECD TGs for safety testing of substances, materials and products. By developing, commenting and contributing as an expert, scientists can have a major impact toward solving global challenges. This could set scientists on the path toward bringing their research toward having regulatory impact.

## Footnotes

^1^https://www.oecd.org/en/about/programmes/oecd-programme-on-chemical-safety-and-biosafety.html (Consulted in May 2025).

^2^https://www.iso.org (Consulted in May 2025).

^3^https://www.oecd.org/en/about/members-partners.html (Consulted in May 2025).

^4^https://ec.europa.eu/research/participants/documents/downloadPublic?documentIds=080166e504772924&appId=PPGMS (Consulted in May 2025).

^5^https://www.oecd.org/en/topics/sub-issues/testing-of-chemicals/test-guidelines.html (Consulted in May 2025).

^6^https://www.oecd.org/content/dam/oecd/en/topics/policy-sub-issues/testing-of-chemicals/work-plan-test-guidelines-2024.pdf (Consulted in May 2025).

^7^https://www.iso.org/obp/ui (Consulted in May 2025).

^8^https://www.astm.org/get-involved/technical-committees/work-items-full-list (Consulted in May 2025).

^9^http://www.vamas.org/ (Consulted in May 2025).

^10^https://cordis.europa.eu/project/id/814401 (Consulted in May 2025).

^11^https://cordis.europa.eu/project/id/885931 (Consulted in May 2025).

^12^https://www.oecd.org/content/dam/oecd/en/topics/policy-sub-issues/testing-of-chemicals/national-coordinators-of-test-guideline-programme.pdf (Consulted in May 2025).

^13^https://malta-initiative.org (Consulted in May 2025).

^14^https://testguideline-development.org/ (Consulted in May 2025).

^15^https://www.nist.gov/srm (Consulted in May 2025).

^16^https://www.comar.bam.de/ (Consulted in May 2025).

^17^https://www.lgcstandards.com/PL/en (Consulted in May 2025).

^18^https://crm.jrc.ec.europa.eu/ (Consulted in May 2025).

^19^https://joint-research-centre.ec.europa.eu/scientific-tools-and-databases/jrc-nanomaterials-repository_en (Consulted in May 2025).

^20^https://ed-pepper.eu/ (Consulted in May 2025).

^21^https://testguideline-development.org/useful-resources#White-paper (Consulted in May 2025).

^22^https://testguideline-development.org/useful-resources#Workshops-webinars (Consulted in May 2025).

^23^https://macrame-project.eu/macrame-meetings-workshops/ (Consulted in May 2025).

^24^https://testguideline-development.org/useful-resources#Introduction-OECD-standardization (Consulted in May 2025).

^25^2024 Work plan of the OECD Test Guidelines programme This work plan is yearly updated around July. https://www.oecd.org/content/dam/oecd/en/topics/policy-sub-issues/testing-of-chemicals/work-plan-test-guidelines-2024.pdf (Consulted in May 2025).

^26^https://www.oecd.org/en/topics/sub-issues/testing-of-chemicals/omics-technologies-chemical-testing.html (Consulted in May 2025).

## References

[B1] Caldeira C. Farcal L. R. Garmendia Aguirre I. Mancini L. Tosches D. Amelio A.. (2022). Safe and Sustainable by Design Chemicals and Materials: Framework for the Definition of Criteria and Evaluation Procedure for Chemicals and Materials. Luxembourg: Publications Office of the European Union.

[B2] EC (2008). Council Regulation (EC) No 440/2008 of 30 May 2008 Laying Down Test Methods Pursuant to Regulation (EC) No 1907/2006 of the European Parliament and of the Council on the Registration, Evaluation, Authorisation and Restriction of Chemicals (REACH). Luxembourg: Publications Office of the European Union, 1–739.

[B3] EC (2012). Regulation (EU) No 1025/2012 of the European Parliament and of the Council of 25 October 2012 on European Standardisation, Amending Council Directives 89/686/E EC and 93/15/E EC and Directives 94/9/EC, 94/25/EC, 95/16/EC, 97/23/EC, 98/34/EC, 2004/22/EC, 2007/23/EC, 2009/23/EC and 2009/105/EC of the European Parliament and of the Council and repealing Council Decision 87/95/EECand Decision No 1673/2006/EC of the European Parliament and of the Council. Luxembourg: Publications Office of the European Union, 12–33.

[B4] EC (2020). Communication From the Commission to the European Parliament, the Council and the European Economic and Social Committee and the Committee of the Regions - Chemicals Strategy for Sustainability Towards a Toxic-Free Environment. COM(2020) 667 Final. Brussels: European Commission.

[B5] EC (2024). Communication from the Commission to the European Parliament, the Council, the European Economic and Social Committee and the Committee of the Regions - Advanced Materials for Industrial Leadership. COM(2024)98 Final. Brussels: European Commission.

[B6] Friedrichs S. Seitz C. Bleeker E. Vandebriel R. Ouhajji S. Duistermaat E.. (2025). MACRAMÉ—Advanced characterisation methodologies to assess and predict the health and environmental risks of advanced materials. Comput. Struct. Biotechnol. J. 29, 95–109. 10.1016/j.csbj.2025.03.032 40236833 PMC11997261

[B7] Halamoda Kenzaoui B. Box H. Van Elk M. Gaitan S. Geertsma R. Gainza Lafuente E.. (2019). Anticipation of Regulatory Needs for Nanotechnology-Enabled Health Products, EUR 29919 EN. Luxembourg: Publications Office of the European Union. 10.2760/596822

[B8] Heunisch E. Cassee F. Bleeker E. Kuhlbusch T. Gonzalez M. (2022). Status Report: Development or Revisions of OECD Test Guidelines (TG) and Guidance Documents (GD) Applicable for Nanomaterials. EU projects NanoHarmony and NANOMET. Available online at: https://www.oecd.org/en/topics/sub-issues/testing-of-chemicals/nanomet.html (accessed May 20, 2025).

[B9] Malta Initiative (2024). Malta Initiative Priority List. Available online at: https://malta-initiative.org/what/#MI-Priority-List (accessed May 20, 2025).

[B10] OECD (2002). Guidance Document for the Development of OECD Guidelines for Testing of Chemicals, OECD Series on Testing and Assessment, No. 1. Paris: OECD Publishing. 10.1787/9789264077928-en

[B11] OECD (2005). Guidance Document on the Validation and International Acceptance of New or Updated Test Methods for Hazard Assessment, OECD Series on Testing and Assessment, No. 34. Paris: OECD Publishing. 10.1787/e1f1244b-en

[B12] OECD (2017). Guidance Document for Describing Non-Guideline in Vitro Test Methods, OECD Series on Testing and Assessment, No. 211. Paris: OECD Publishing. 10.1787/9789264274730-en

[B13] OECD (2018). Guidance Document on Good in Vitro Method Practices (GIVIMP), OECD Series on Testing and Assessment, No. 286. Paris: OECD Publishing. 10.1787/9789264304796-en

[B14] OECD (2019). Saving Costs in Chemicals Management: How the OECD Ensures Benefits to Society. Paris: OECD Publishing. 10.1787/9789264311718-en

[B15] OECD (2020). Overview of Concepts and Available Guidance related to Integrated Approaches to Testing and Assessment (IATA), OECD Series on Testing and Assessment, No. 329. Paris: OECD Publishing. 10.1787/cd920ca4-en

[B16] OECD (2021). Guidance Document on the Characterisation, Validation and Reporting of Physiologically Based Kinetic (PBK) Models for Regulatory Purposes, OECD Series on Testing and Assessment, No. 331. Paris: OECD Publishing. 10.1787/d0de241f-en

[B17] Subramanian V. Peijnenburg W. J. Vijver M. G. Blanco C. F. Cucurachi S. Guinée J. B. (2023). Approaches to implement safe by design in early product design through combining risk assessment and Life Cycle Assessment. Chemosphere 311, 137080. 10.1016/j.chemosphere.2022.137080 36328317

[B18] Xie H. Wei X. Zhao J. He L. Wang L. Wang M.. (2022). Size characterization of nanomaterials in environmental and biological matrices through non-electron microscopic techniques. Sci. Total Environ. 835, 155399. 10.1016/j.scitotenv.2022.155399 35472343

